# A perspective current and past modes of inhalation therapy

**DOI:** 10.1111/1751-7915.14419

**Published:** 2024-02-22

**Authors:** José Canto Mangana, Kelsey Aguirre Schilder, José Ignacio Bretones‐Pedrinaci, Ana Rosa Márquez Blesa, Fermín Sánchez de Medina, Olga Martínez‐Augustin, Abdelali Daddaoua

**Affiliations:** ^1^ Department of Biochemistry and Molecular Biology II, Pharmacy School University of Granada Granada Spain; ^2^ Pharmacy Services A.S. Hospital de Poniente de Almería Almería Spain; ^3^ Department of Pharmacology, School of Pharmacy University of Granada Granada Spain; ^4^ Centro de Investigación Biomédica en Red de Enfermedades Hepáticas y Digestivas (CIBERehd) Madrid Spain; ^5^ Instituto de Investigación Biosanitaria (IBS) Granada Spain; ^6^ Institute of Nutrition and Food Technology ‘José Mataix’, Center of Biomedical Research University of Granada Granada Spain

## Abstract

Inhalation is the preferred route of delivery for anti‐asthma and chronic obstructive pulmonary disease (COPD) drugs. The use of this route has demonstrated efficacy in these and other conditions, it offers rapid onset of action, and is associated with minimal systemic exposure, thereby reducing the risk of adverse effects. Therefore, the current brief covers an interesting collection of inhaler action modes, shedding light on their molecular mechanisms and clinical applications for anti‐asthma, COPD and antibacterial inhalation therapy. Hence, not only enriches our understanding of inhalation therapy molecular intricacies but also provides a comprehensive overview of the evolving landscape in clinical and antibacterial inhalation therapy. In doing so, it underscores the pivotal role of microbiology and biotechnology in advancing therapeutic approaches that harness the power of inhalation.

## INTRODUCTION

Inhaling vapours of substances such as incense or aromatic oils, resins and perfumes to alter consciousness or as part of religious ceremonies dates back to ancient cultures. Hence, around 1550 BC, drawings from the Joseph Smith Egyptian papyri revealed that treatments for respiratory problems included not only the consumption of a variety of preparations with plant, animal and mineral products, but also inhaling vapours from incinerated henbane (*Hyoscyamus niger*) leaves. The therapeutic properties of this vapour have been attributed to tropane alkaloids, such as atropine, whose derivatives are still used today in certain inhaler products (Stein & Thiel, 2017). By the end of the 19th century through the 20th century, cigarettes made from *Datura stramonium* and other ingredients such as *Camellia* spp. and *Atropa belladonna* were used to treat asthma and other lung diseases. However, probably the most prominent form of inhalation throughout history has been the smoking of opium (*Papaver somniferum*) by way of pipes or burners. It is documented as early as the second or third millenium BC in China and Sumer and it spreaded promptly to Egypt, Carthage and Europe. The medicinal properties of opium were described later by Avicenna (1025 AD), who showed in his medical encyclopaedia, Canon of Medicine (*Al‐qanun fi altibb*) that opium had analgesic and anti‐diarrhoeal properties, but could be toxic and easily overdosed, advising against its use (Sneader, 2005; Stein & Thiel, 2017). Opium was also used for recreational purposes.

Later, the Indian physicians Charaka and Sushruta (600 BC) described that the leaves of *Datura stramonium* could be used to produce an aerosol to relieve dyspnoea, with an effect reminiscing that of bronchodilators (Sneader, 2005). The information about aerosol therapy from the fall of the Roman Empire to the Industrial Revolution is scarce. Important developments took place in the 18th and 19th centuries related to the first device that could be referred to as an ‘inhaler’, which was devised in the late XVIII century by John Mudge. The patient aspirated for 15–30 min air from a tube with a mouthpiece as it passed through hot liquid in a metallic jar. Humphry Davy first observed the biological properties of nitrous oxide and coined the expression ‘laughing gas’ in 1799. Observing the anaesthetic effect, Davy proposed that the gas could be used for surgical operations, although this was not to occur until half a century later. Thus, nitrous oxide was first used for dentistry and surgery, as an anaesthetic and analgesic in 1844 (Sneader, 2005). Ethyl ether was introduced around the same time, and chloroform shortly thereafter (Rospond et al., 2022). Gas anaesthetics, particularly ether, were also used for recreational purposes, and from the 1940s on a number of solvents were similarly incorporated to this practice, including gasoline, paint, or nail polish remover, among others.

The first nebulisers were developed in the middle of the 19th century. The best known is the ‘Pulverisateur’, in which a pump with a handle forces the liquid through a nozzle that nebulises it (Ito, 2022). Between 1930 and 1950, hand‐held nebulisers and the first electric nebulisers were developed to deliver active ingredients, such as epinephrine, into the airways of asthmatic patients. In 1967, the first dry powder inhaler (Spinhaler) was developed.

Different drugs can be administered successfully and with improved efficacy and toxicity via inhalers, particularly bronchodilators and corticosteroids. In addition, there is growing interest in the application of antifungals, antibiotics, and antivirals (Ito, 2022). Regardless of age, the inhalation pathway is considered the route of choice for the treatment of asthma and chronic obstructive pulmonary disease (COPD).

This brief report describes the mechanistic aspects underlying the drug composition of the most commonly used inhalation devices (Table 1) as well as other inhalers used against bacterial, fungal, and viral infections (Table 2) (Spacova et al., 2021). Currently, dry powder inhalers (DPIs) and pressurized metered dose inhalers (pMDIs) are the most widely used devices. DPIs are used to administer medication in the form of powder in suspension while MDIs use a propellant to deliver a metered dose of medication as a fine mist or aerosol. Another type, introduced in 2003, is the soft mist inhaler (SMI), which generates a low speed, small particle aerosol without the help of a propellant. It requires no inspiratory effort or coordination, thereby increasing its effectiveness. Moreover, there are other forms to administer antibacterial medication to the lungs, namely through‐aerosolized solutions or liposomal solution forms.

**TABLE 1:** Inhaler identification, action mode and clinical application.

Drug class	Drug name	Formulation	Drug combination	Propellant^a^	Mode of action	Clinical application
Corticosteroids	Beclomethasone dipropionate	DPI‐capsule	Glycopyrronium bromide + formoterol fumarate		Binds to the glucocorticoid receptor, inhibiting proinflammatory signals, and promoting anti‐inflammatory signals	COPD ASTHMA
			Formoterol fumarate			
		pMDI‐extrafine solution		Norflurane		
	Budesonide	DPI‐multidose				
		pMDI	Formoterol fumarate	Apaflurane		
		DPI‐multidose				
	Fluticasone propionate	DPI‐blister				
		pMDI‐suspension	Formoterol fumarate	Norflurane		
	Fluticasone furoate	DPI‐blister				
	Mometasone furoate	DPI‐multidose				
	Ciclesonide	pMDI‐extrafine solution		Norflurane		
SABA	Salbutamol	pMDI‐suspension		Norflurane	Agonist β2‐receptors	COPD ASTHMA
		DPI‐multidose				
		Solution	Ipratropium			
	Terbutaline	DPI				
LABA/VLABA	Formoterol fumarate	pMDI‐extrafine solution	Beclomethasone dipropionate	Norflurane		
		DPI‐multidose				
			Budesonide			
			Aclidinium			
	Salmeterol xinafoate	DPI‐blister	Fluticasone propionate			
		pMDI‐solution		Norflurane		
	Vilanterol	DPI‐blister	Umeclidinium bromide			
			Fluticasone propionate + umeclidinium bromide			
	Indacaterol maleate	DPI‐CAPSULE	Glycopyrronium bromide			
	Olodaterol	SMI	Tiotropium			COPD
SAMA	Ipratropium bromide	Solution	Salbutamol		Antagonist muscarinic receptors (M1, M2, M3)	COPD ASTHMA
		pMDI		Tetrafluoromethane		
LAMA	Aclidinium bromide	DPI‐multidose	Formoterol fumarate		Antagonist muscarinic receptors (M1, M2, M3)	COPD
	Umeclidinium bromide	DPI‐blister	Vilanterol			
			Vilanterol + fluticasone propionate			
	Tiotropium	SMI	Olodaterol			COPD ASTHMA
		DPI‐capsule				
	Glycopyrronium bromide	DPI‐capsule	Indacaterol maleate			
			Beclomethasone dipropionate + formoterol fumarate			

Abbreviations: COPD, chronic obstructive pulmonary disease; CQ, cystic fibrosis; DPI, dry powder inhaler; pMDI, pressurized metered dose inhaler; SMI, soft mist inhaler.

^a^ Propellants: Only in pMDI device.

**TABLE 2:** Others inhaler identification, action mode and clinical application.

Drug class	Drug name	Formulation	Excipient used	Mode of action	Clinical application
Antivirals	Remdesivir	Dry powder inhaler	Dipalmitoylphosphatidylcholine (DPPC), captisol, mannitol, lactosa, L‐leucine	RdRp inhibitor	Coronavirus disease pneumonia. SARS‐CoV‐2
		Liposomal solution for nebulisation	Cholesterol, trehalose		
	Triazavirin	Solution for nebulisation		Inhibitor RNA synthesis	
	Hydroxychloroquine sulphate	Dry powder inhaler		Inhibition through interference in the endocytic pathway	
				Inhibition through blockade of sialic acid receptors	
		Solution for nebulisation	Sterile water	Inhibition through restriction of pH mediated S protein cleavage at the ACE2 binding site	
				Inhibition through prevention of cytokine storm	
	Interferon‐α2b	Solution for nebulisation	Sterile water	Inhibition protein synthesis. Inactivation of viral RNA, and enhancement of phagocytic and cytotoxic mechanisms	
	Zanamivir	Dry powder inhaler	Lactosa	Inhibition of virus neuraminidase	Prevention influenza A/B
	Inavir				
	Ribavirin	Solution for nebulisation	Sterile water	Nucleoside antimetabolite	Respiratory syncytial virus (RSV) pneumonia
Antibiotics	Colistimethate	Dry powder inhaler		Induce changes in the permeability of the cell membrane. Bind anionic lipopolysaccharide	Cystic fibrosis. *Pseudomonas* spp.
		Solution for nebulisation	Sterile water		
	Levofloxacin	Solution for nebulisation	Sterile water, magnesium chloride	Inhibits of bacterial topoisomerase IV and DNA gyrase	
	Tobramycin	Dry powder inhaler	Distearoylphosphatidylcholine, calcium chloride	Bind 30S and 50S ribosome, preventing formation of the 70S complex. Inhibits protein synthesis	
		Solution for nebulisation	Sterile water		
	Amikacin	Liposomal solution for nebulisation	Sterile water, DPPC, cholesterol		Pulmonary disease. *M. avium* complex *M. abscessus*
		Solution for nebulisation	Sterile water		
	Gentamicin	Solution for nebulisation	Physiological saline solution		Cystic fibrosis. *Pseudomonas* spp.
	Ampicilin		Sterile water	Inhibition of bacterial cell wall synthesis. Bind to PBP	
	Cefotaxime				
	Ceftazidime				
	Imipenem/cilastatin		Physiological saline solution		
	Meropenem				
	Aztreonam lysine		Sterile water, L‐lysine		
	Vancomycin		Physiological saline solution	Inhibition of bacterial cell wall synthesis. Bind to acyl‐D‐ala‐D‐ala	Cystic fibrosis. Methicillin‐resistant *Staphylococcus aureus*
Antifungals	Amphotericin B liposomal	Liposomal solution for nebulisation	Sterile water, DPPC, cholesterol	Formation of ion channels in cell membrane. Bind to ergosterol	Pneumonia*. Candida* spp. *Aspergillus* spp.
	Amphotericin B deoxycholate	Solution for nebulisation	Sterile water		
	Voriconazole	Cyclodextrin‐solution for nebulisation	Hydroxipropyl betadex	Inhibits ergosterol synthesis. Bind to 14‐alpha sterol demethylase	
		Dry powder inhaler	Polyvinyl pyrrolidone K‐12		
Probiotics and postbiotics experimentation	*Corynebacterium pseudodiphtheriticum* 090104, *Dolosigranulum pigrum* 040417	Suspension for nasally administration	Phosphate buffer saline	Modulate lung innate immune response related by IFN‐β, IFN‐γ, (IL)‐10	Increased resistance to respiratory syncytial virus and *Streptococcus pneumoniae* superinfection in a mouse model
	*Lacticaseibacillus rhamnosus GG*			Enhanced natural killer (NK) cell activation in the lungs and modulate innate immune response by IL‐1β, TNF	Lower clinical symptom and increased survival in a mouse model of H1N1 influenza virus infection
	*L. rhamnosus* GG			Induction of a transcriptional type I IFN immune genes for an efficient antiviral response	Improved survival and delayed mortality in a neonatal mouse model of influenza infection
	*Lactiplantibacillus pentosus* S‐PT84	Solution for nasally administration	Sterile water	Increased NK cell activity, IFN‐α and IL‐12 concentrations in the lung	Dose‐dependent increase in survival rates in a mouse model of mouse‐adapted H1N1 influenza virus infection
	*Limosilactobacillus fermentum* CJL‐112	Suspension for nasally administration	Sterile saline (0.85% NaCl)	Activation of T helper 1 responses reflected in upregulation of lung IFN‐γ and IL‐12	Protection against influenza A/NWS/33 (H1N1) infection in a mouse model
	*Limosilactobacillus reuteri* F275		Albumin, phosphate buffer saline	Promote neutrophil influx into the lungs associated with increase in proinflammatory cytokines and chemokines	Robust and sustained resistance to challenge with the pneumonia virus of mice
	*Lactiplantibacillus plantarum* NC8 expressing the NP‐M1‐Dcpep antigen from avian influenza virus H9N2		Phosphate buffer saline	Protective immune response linked with induction of mucosal IgA and systemic IgG	Protection against H9N2 avian influenza virus challenge in vaccinated chickens
	*L. casei* BLS‐S8 displaying the *SARS‐CoV* spike protein			Induction of neutralizing antibody, production of antigen‐specific mucosal IgA	Production of protective antibodies versus SARS‐CoV in mice
	Cell wall of *D. pigrum* 040417			Reduction in lung injury markers; increase in lung levels of IFN‐γ and IFN‐β	Improved response against RSV and S. pneumoniae superinfection in a mouse model
	Non‐viable and viable *L. rhamnosus* CRL1505			Increase in systemic (serum) and local (airways) regulatory cytokine IL‐10 and type I interferons (IFN‐β and IFN‐γ)	Improved infection outcomes through reduction of pulmonary damage and lung viral loads in a mouse model of influenza
	Peptidoglycan of *L. rhamnosus* CRL1505 or live bacteria			Modulation of lung innate immune responses through generation of activated CD11c + SiglecF+ alveolar macrophages	Improved resistance to primary RSV infection and secondary pneumococcal pneumonia
	Poly‐gamma glutamate synthesized by *Bacillus subtilis* var. chungkookjang KCTC 0697BP			Enhanced antiviral cytokine production (especially IFN‐β and IL‐12), increased activation of NK cells, and higher levels of cytotoxic T lymphocyte activity against influenza	Improved survival in mice infected with H1N1 influenza A virus

### Drugs/inhalers development

Table 1 collects the most successful and most clinically used drugs/inhalers. COPD and asthma are the main conditions being treated with this type of devices (Sorino et al., 2020; Zhou et al., 2015). Beta 2 agonists stimulate beta‐2 receptors in the lungs to relax the smooth muscle cells in the airway, allowing it to open and therefore making it easier to breathe. Short‐Acting Beta‐Agonists (SABA, with a duration of effect of 4–6 h) most commonly used are usually administered via inhalation using a metered‐dose inhaler or a nebulizer and include salbutamol (Ventolin®, Proventil®) and terbutaline (Terbasmin®). Long‐Acting Beta‐Agonists (LABA, ~12 h effect) include salmeterol (Serevent®) and formoterol (Foradil®), while Very Long‐Acting Beta‐Agonists (VLABA, 24 h effect), with the longest duration of action, include indacaterol (Breezhaler®) and vilanterol (Ellipta®). Beta‐agonists, particularly LABA and VLABA, are often formulated in combination with an inhaled corticosteroid (ICS) (Table 1).

SAMA stands for Short‐Acting Muscarinic Antagonist (typically lasting 4 to 6 h) and LAMA for Long‐Acting Muscarinig Antagonist (effect lasts for 12 h or more) (Table 1). Both block the action of acetylcholine, a neurotransmitter that causes constriction of the airways (Sorino et al., 2020; Zhou et al., 2015). The most commonly used SAMA and LAMA include ipratropium bromide (Atrovent®) and tiotropium (Spiriva®), aclidinium (Tudorza®) or umeclidinium (Incruse®, Ellipta®), respectively (Terry & Dhand, 2020).

### Other inhaled medications

Biotechnology and bioengineering techniques play a significant role in the design and development of inhalers directed to the management of respiratory infections by the inhalation route. Hence, there is increased interest among researchers in developing innovative inhalers capable of delivering bacterial‐based therapies. The challenge in this approach is to deliver antimicrobial drugs efficiently, ensuring effective release within the respiratory tract and reducing adverse effects associated with systemic exposure (Ito, 2022; Zhou et al., 2015). Currently, in cystic fibrosis patients with *Pseudomonas* spp. or *Staphylococcus aureus* chronic infection antibiotics may be delivered by dry powder inhalation or nebulisation of antibiotic, such as aztreonam lysine (O'Sullivan et al., 2010) or colistimethate (Vardakas et al., 2018), to inhibit bacterial cell wall synthesis or to induce changes in the permeability of the bacterial membrane, respectively (Table 2). In addition, one of the most promising antibacterial strategies is the use Nebulizer eFlow® with levofloxacin to inhibit bacterial topoisomerase IV and DNA gyrase, or the dry powder inhaler with tobramycin, to block the protein synthesis by binding the subunits 30S and 50S of ribosome and prevent the formation of the 70S complex (Table 2) (Bassetti et al., 2020).

Likewise, *Aspergillus* infections in patients with cystic fibrosis, severe asthma and COPD are undertreated due to the low benefit–risk ratio of available treatments. Inhaled antifungals can be delivered directly to the site of infection in the lungs, potentially offering more targeted and effective treatment by inhibiting the growth and spread of the fungus responsible for the infection. Thus, inhaled itraconazole can be safely used for the treatment of pulmonary fungal infections in patients concurrently treated with drugs that are metabolized by CYP3A4 due to the inhibitory function (Mackenzie et al., 2023) as well as in *Aspergillus*‐related infection issues (Spacova et al., 2021). Likewise, liposomal amphotericin B, which acts by the formation of ion channels in ergosterol containing membranes, was used as solution for nebulisation against *Candida* spp. and prophylaxis against invasive pulmonary aspergillosis in patients with pneumonia (Table 2) (Mackenzie et al., 2023; Spacova et al., 2021). Recently, the coronavirus (COVID‐19) pandemic has caused millions of deaths with an extreme impact on the world. This circumstance has created the need to rapidly develop new and effective ways to control and treat the virus, in support of the already available COVID‐19 vaccines, including inhalation therapies (Saha et al., 2022). The efficacy of nitric oxide (Winchester et al., 2021), or aerosol treatments, such as N‐acetylcysteine (Panahi et al., 2023), Triazavirin or Remdesivir (Mechineni et al., 2021; Vermillion et al., 2022), is currently being evaluated for the treatment of COVID‐19 (Table 2). Remdesivir, first developed and tested during the Ebola epidemic and other viral diseases (Mechineni et al., 2021) and Triazavirin are both RNA‐dependent RNA polymerase inhibitors (RdRp) which play an essential role in viral transcription and maturation inside the host (Mechineni et al., 2021). Likewise, inhaled drug therapy has been applied in COVID‐19 pneumonia (Brüssow, 2021), namely Hydroxychloroquine sulphate and Interferon‐α2b. Administration of neuraminidase inhibitors as dry powder inhalers to prevent influenza A/B infection is another active area (Zanamivir, Laninamivir) (Anderson et al., 2022; Kanan et al., 2023) (Table 2). Finally, Favipiravir® and Ribavirin® are other examples of this approach.

Nevertheless, Pre‐clinical trials have shown the inhaled aerosol vaccine is far more effective at inducing protective immune responses than traditional injections, partly because it targets the lungs and upper airways where viruses first enter the body, providing long‐lasting protection against respiratory infections. In fact, Sam Afkhami et al., 2022 have developed in animals a next‐generation ChAd‐vectored trivalent COVID‐19 vaccine which demonstrated the superiority of a single‐dose intranasal immunization in inducing the tripartite respiratory mucosal immunity against both ancestral and variant strains of SARS‐CoV‐2. However, whether the same holds true in humans remains to be investigated.

### A bright future for inhalers

One of the latest advancements in the field is the application of nanopharmaceuticals, i.e. pharmaceutical formulations or drug delivery systems that incorporate nanotechnology for improved drug efficacy by optimizing drug pharmacokinetics, enhancing cellular uptake, overcoming biological barriers, and minimizing systemic side effects. In principle, nanopharmaceuticals can be used to develop inhalable drug formulations that are more effective and efficient for the treatment of respiratory diseases such as asthma, cystic fibrosis or COPD (Barthold et al., 2023) using liposome formulations. Liposomes are nanosized lipid vesicles, which can encapsulate drugs and deliver them to the lungs. Similarly, polymeric nanoparticles, solid lipid nanoparticles, and dendrimers are also being explored as inhalable drug carriers (Barthold et al., 2023). However, research in the field of probiotics, postbiotics, and their potential use in inhalers treatments, continues to expand (Spacova et al., 2023). The majority have been developed as suspension for nasal administration against SARS‐CoV, influenza ssp, streptococcus and pneumococcal infection (Table 2). Nonetheless, while nanopharmaceuticals hold great promise, their development and regulatory approval require extensive research, testing, and evaluation to ensure their safety and efficacy.

## CONCLUSION

Inhalation is the preferred route of delivery for anti‐asthma and COPD drugs. The use of this route has demonstrated efficacy in these and other conditions, it offers rapid onset of action, and is associated with minimal systemic exposure, thereby reducing the risk of adverse effects. Nevertheless, it has some drawbacks. pMDIs and DPIs require an inspiratory flow of 20–60 L/min, depending on the device, which may pose a difficulty for certain patients. Use requires adequate technique that may not be feasible for all. In many cases inhaled particles deposit in the oropharynx, resulting in dose loss or adverse effects in the case of corticosteroids. Moisture can alter the particles in some devices. Healthcare professionals should be familiar with the different inhalation devices and their administration technique for good control administration. Although several improvements in pMDIs such as a changes in the propellant and actuation have resulted in improvements in lung deposition, many dry powder inhalers (DPIs) are easier to use, as are soft mist inhalers. Beta 2 agonists, antimuscarinic agents and corticosteroids are the most widely used drugs in inhalatory therapy. Likewise, new advances may change the landscape of inhalational antibiotic and antiviral therapies against particular infections; hence, their application could be extended in the future for several anti‐infectious treatments.

## AUTHOR CONTRIBUTIONS

**José Canto Mangana:** Investigation (equal); methodology (equal). **Kelsey Aguirre Schilder:** Investigation (equal). **José Ignacio Bretones‐Pedrinaci:** Investigation (equal); methodology (equal). **Ana Rosa Márquez Blesa:** Investigation (equal). **Fermín Sánchez de Medina:** Validation (equal); writing – original draft (equal). **Olga Martínez‐Augustin:** Validation (equal); writing – original draft (equal). **Abdelali Daddaoua:** Conceptualization (equal); methodology (equal); supervision (equal); writing – original draft (equal); writing – review and editing (equal).

## FUNDING INFORMATION

No funding information provided.

## CONFLICT OF INTEREST STATEMENT

The authors declare that they have no known competing financial interests or conflict that could have appeared to influence the work reported in this document.
